# Who are Your Joneses? Socio-Specific Income Inequality and Trust

**DOI:** 10.1007/s11205-016-1460-9

**Published:** 2016-10-03

**Authors:** Fabian Stephany

**Affiliations:** University of Cambridge and Vienna University of Economics and Business, Vienna, Austria

**Keywords:** Income inequality, Trust, Stratification, Perception

## Abstract

Trust is a good approach to explain the functioning of markets, institutions or society as a whole. It is a key element in almost every commercial transaction over time and might be one of the main explanations of economic success and development. Trust diminishes the more we perceive others to have economically different living realities. In most of the relevant contributions, scholars have taken a macro perspective on the inequality-trust linkage, with an aggregation of both trust and inequality on a country level. However, patterns of within-country inequality and possibly influential determinants, such as perception and socioeconomic reference, remained undetected. This paper offers the opportunity to look at the interplay between inequality and trust at a more refined level. A measure of (generalized) trust emerges from ESS 5 survey which asks “...*generally speaking, would you say that most people can be trusted, or that you can’t be too careful in dealing with people?*”. With the use of 2009 EU-SILC data, measurements of income inequality are developed for age-specific groups of society in 22 countries. A sizable variation in inequality measures can be noticed. Even in low inequality countries, like Sweden, income imbalances within certain age groups have the potential to undermine social trust.

## Introduction

The undesirable outcomes linked to high levels of income inequality^1^ are numerous. Among several, there are poor educational performance (Kawachi et al. 1997), violence (Patterson 1991), low levels of wellbeing (Alesina et al. 2004) and health (Wilkinson 2006), as well as slow economic growth (Barro 2000) or low provision rates of social goods (Pickett and Wilkinson 2009). It is argued that income inequality has a destructive effect on civic cooperation, since economic stratification leads to low levels of trust (Pickett and Wilkinson 2009). In addition, it has been shown that social trust is indeed negatively affected by income inequality (Leigh 2006b; Uslaner and Brown 2005; Gustavsson and Jordahl 2008). Inequality is believed to reduce (generalized) trust since it stratifies society into distinct groups which are unable to associate with each other anymore. The link between inequality and trust has so far been examined mainly by looking at stratification and the society as a whole.

The radius of our social interactions, however, has been shown to be a very relevant in how much we trust others (Knack and Keefer 1997; Delhey et al. 2011). This study therefore argues that perception of inequality is mainly made by socio-economic benchmarking. Age- and education-specific measures of income inequality are calculated with the use of European income data. Together with individual characteristics, the inequality measures are combined in a multilevel ordered logit model in order to better explain patterns of generalized trust. General income inequality might not capture the age-specific imbalances. By national Gini, Sweden, for example, has one of the lowest levels of income inequality in Europe. However, income imbalances for the five oldest age cohorts (75–80 years-old) reach a level of 0.33, which is just about the average of national inequality in Europe.

The results of the study illustrate that income imbalances within European societies vary to a sizable extent across age and education. In some high inequality countries, like Bulgaria or Portugal, inequality volatility across age is particularly large. At the same time we see that age-specific income inequality, just as “standard” inequality is related negatively to trust, controlling for relevant personal characteristics. Generalized trust decreases with high levels of general and age-specific income inequality. The refinement of income inequality for different age-groups does not challenge the inequality-trust linkage, but it adds more explanatory power compared to the use of general income inequality. The results show that some of the variation of generalized trust is captured by the variance of income inequality across age. Age-specific inequality is rather a complement than a substitute to national inequality. The interaction between general income inequality and age-specific inequality shows a negative association with trust, too. This could be explained by the idea that age-specific inequality matters even more if observed in an environment of overall high levels of inequality. Though, a direction of causality, from inequality to trust, is assumed in this work. An opposite direction of cause and effect could be immagined in some circumstances. In order to approach the question of causality, future research should include a longditudinal dimension.

The next section discusses the theoretical concepts of trust and inequality relevant to the analysis. Sections 3 and 4 of this work present the data and the method, used to entangle relations between age-specific inequality and trust. Section 5 summarizes the results and Sect. 6 concludes.

## Trust and Inequality

### About Generalized Trust

In order to capture the concept of social or interpersonal trust, the generalized trust question—as applied in this study—has become the most commonly used approximation in empirical analyses. The use of one single survey question in order to picture trust has been criticized and even Putnam (1995), strongly convinced of the importance of trust for social capital has expressed his displeasure about the problem: “since trust is so central to the theory of social capital, it would be desirable to have strong behavioral indicators of trends in social trust or misanthropy. I have discovered no such behavioral measures.”

The most common criticism to the generalized trust question, which asks for the trust in *most people* addresses its vague, abstract, and subjective character. It is argued that the term *most people* might be interpreted quite differently from individual to individual. The idea to whom or which group of people one refers when asked about generalized trust is known in the literature as *the radius problem*. What is the radius of social interactions to which those *most people* belong to? Surely, as interpersonal trust, like human intelligence for example, is a non-tangible concept, approaches to make it measurable will always have to face some shortcomings. The following considerations should underline why the generalized trust question is a valid choice when it comes to catch hold of interpersonal trust.

Delhey et al. (2011) have shown that people distinguish at least in two different set of social interactions and therefore two distinct groups of trust, in-group and out-group trust. In their cross country analysis, they state that *most people* in standard questions most commonly refer to out-groups. They acknowledge the subjectivity of the radius of *most people*, but point out that the variation is driven by cultural differences rather than by individual perception. The radius is quite narrow in Confucian countries, wider in wealthy countries, and—in this particular case—relatively comparable across European societies.

In addition, Knack and Keefer (1997), argue that the actual strength of the generalized trust question lies in its ambiguity. Since it potentially includes a large radius of individuals—not only friends and family members—the generalized trust question mixes two different concepts. On the one hand, how much one trusts in people who are not friends and relatives, and, on the other hand, how often an individual encounters with such persons. In low-trust environments, transaction with close friends and family members is much more common than in high-trust environments. If the respondents to the *most people* question consider the individuals they interact with, the variation of the trust measure should be reduced, which makes it a more reliable measure in explaining potential outcomes like growth or civic participation. In their empirical analysis, the centered pattern of generalized trust is confirmed.

Lastly, a study by Glaeser et al. (2000) with the promising title “Measuring Trust” defends the validity of the generalized trust measure by comparing it with trust and trustworthiness items from two experiments. Generalized trust—stemming from the controversial question—is shown to be a very good predictor for trusting behaviour in the experiments. Furthermore, the researchers point out that in their experiments the answer to the generalized trust question could actually be interpreted as a signal for the own level of trustworthiness and not so much as a representation of mutual confidence in others.

### Stratification and Perception

The essential argument why inequality reduces generalized trust is that differences between individuals are too large to trust each other. Two major lines of reasoning are relevant in this respect. On the one hand, scholars like Tumin (1953) have shown that generalized trust diminishes the more economically stratified a society is. Distinct spheres of everyday life emerge (segregated housing, public versus private schooling etc.) within which understanding and trust for “outsiders” fade away. This association is described as the *stratification effect*. On the other hand, claims have been made (Brockner 1996) that perceptions and believes about the distribution of resources and how they should ideally be allocated determine our trust in others by the mechanism of the so-called *perception effect* (Uslaner and Brown 2005). Previous studies have claimed to capture the effects of both stratification and perception with the use of one inequality indicator. If one believes that the comparison with others is key to understand how inequality influences trust, the concept of economic reference groups should be described accordingly. Instead to comparing one’s own economic well-being to the rest of the society, it is much more likely, that we chose a reference group to which a meaningful comparison is possible, in the first place. For an evaluation of the *perception effect* of inequality, *“our family Jones*”,^2^ to use the term of social class benchmarking, still needs to be defined.

### The Inequality Within

The inequality-trust linkage has so far been examined only by looking at stratification and the society as a whole, while it can be assumed that personal characteristics, as age and education, play an important role in the deeper understanding of how income distributions are associated with generalized trust, for mainly two reasons. First, our understanding of justice strongly determines to what extent inequality might affect our trust. At the same time, our views on equity and justice change with experience and education during the life course. In addition, it can be questioned that the extent to which we are exposed to an assumed *stratification effect* is the same in all stages of our life. The degree of interaction with individuals from other social groups might be different for a student compared to a senior employee or a pensioner. It is therefore necessary to claim that the association between inequality and trust changes as we age and gain knowledge. Second, scholars advocating the *perception effect* have rarely specified the corresponding reference group. If personal frustration about unjust allocation and distribution of economic resources influences our level of trust (Pickett and Wilkinson 2009), then the question arises to whom we actually compare ourselves. A 20 year-old student would perceive the economic success of a 40 year-old manager rather as something aspiring than something unjust. One probable assumption is that the perception of inequality within our age-specific socio-economic reference groups strongly determines our level of generalized trust.

### Life-Cycle Inequality

Only in most recent efforts, attention has been drawn to the shortcomings of comparing income and wealth of individuals in different stages of their life-cycle and economic career, such as in Bönke et al. (2011). The authors show that earnings distributions for German men vary substantially during their life course. Their research on lifetime inequality, in the example of Germany, has shown that earnings inequality develops in a U-shaped pattern over age. Inequality is relatively high for cohorts below the age of twenty, because many have not yet entered the labour market. After that, inequality declines to a minimum level, which is located around the age of thirty. In the following period rising inequality of annual earnings begins. At the age of sixty and above the income distribution has reached the same level of Gini, as the distribution of the 20-years old. In accordance with this known pattern of income inequality over age, the assembly of distinct age-specific Gini indexes is advisable.

### Perception Matters

“Between a condition of objective inequality and the response of a disadvantaged person lie the perceptions, evaluations, expectations—in short, the psyche—of the individual.”

This quote by Dahl (1971, 95) well summarizes the relevance of subjectivity in the analyses of inequality studies in general. The need of a multi-dimensional measure of inequality becomes evident once we consider the theoretical relationship between inequality and trust. One of the main arguments for the inequality-trust-linkage is that with rising imbalances in economic resources, people do not perceive to share a common fate or meet the same challenges in life. Osberg and Smeeding (2006), in this context, refer to the fact that inequality perception does not necessarily mirror actual inequalities, since individuals do not dispose over the information about the entire income distribution. They rely on various sources, like the media, political agendas, and most importantly, one might say, the comparison of the own standard of living with others.

Conditions linked to generalized trust can be influenced by people’s perception of inequality. Pickett and Wilkinson (2009), for example, suggest that inequality results in jealousy and envy by the less fortunate. To which extent we have optimism in the future, which is indeed important for trust (Delhey and Newton 2005; Rothstein and Uslaner 2005), might be harmed by high perceived inequality. Other scholars (Uslaner 2002; Gustavsson and Jordahl 2008), have argued that safeguards of trust, like egalitarian values, are eroded by increased perceived inequality. It is a cumbersome task for researchers to capture the effects of perceived inequality. This study argues, however, that perception of inequality is mainly made by socio-economic benchmarking. Therefore, a more refined measure of reference-specific inequality, for example, the distribution of income within distinct groups of similar age and education, brings us one step closer to capturing the impact of perceived inequality. In this study, age-specific measures of income inequality are applied in order to better explain patterns of generalized trust.

### The Problem of Mutual Dependence

When discussing plausible determinants of trust, quickly, the question about mutual effects becomes apparent. In some cases, the direction of cause and effect is debatable. Many scholars argue that civic engagement or economic growth is fostered by generalized trust, though one could easily argue that a working civil society and economic prosperity lay the ground for people trusting each other. In fact, for the case of civic participation and generalized trust, an ongoing reciprocative relation has become the consensus in the literature (van Ingen and Bekkers 2015). For other features, however, it is much more plausible to argue for a one-sided direction of influence. For the example of religious opinions, it might be true that our denomination has an impact on how much we trust each other, while it is hard to imagine an effect in the opposite direction. The present investigation is based on the assumption that trust is partly determined by income inequality and the perception of it. Still, future investigations shall be encouraged to include a temporal components so as to allow more determined statements about the cause and effect of generalized trust.

## Data

In order to assess the influence of income inequalities on individual (generalized) trust, two datasets are employed; the European Union Statistics on Income and Living Conditions (EU-SILC) and the European Social Survey (ESS 5) of 2010.

**Fig. 1:**
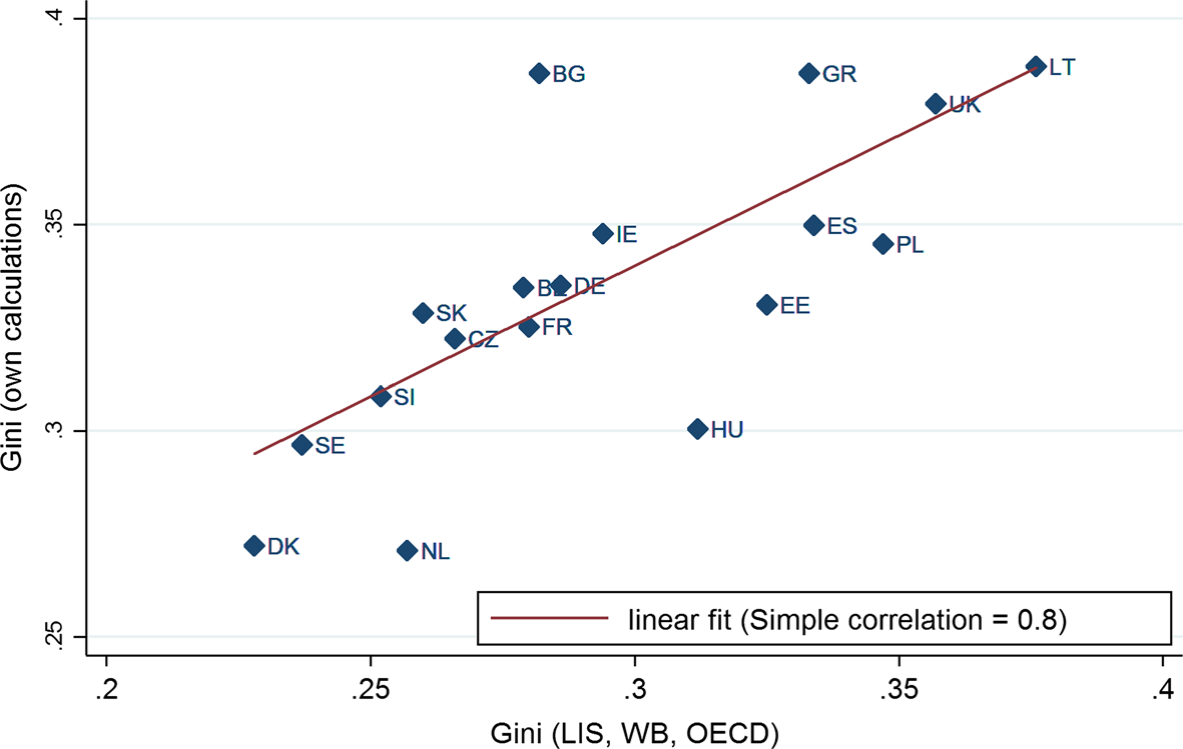
Gini versus own calculations: the official inequality measures and own calculations of Gini coincide. *Source* EU-SILC 2009, ESS 5, and own calculations

The EU-SILC micro data provides us with the information of the monthly gross household income on 32.377 households in 30 European countries. Additionally, the age and educational attainment of the person providing the households accommodation is reported. With the use of the Gini formula derived by Milanovic (1997), age-specific Gini coefficients are calculated for each country. In Fig. 1 the Gini coefficients of our calculations are plotted against the official 2010 net income inequality measures issued by the Luxembourg Income Study database, the OECD and the Worldbank.

The figure supports the reliability of the EU-SILC income data by comparing the calculated country Ginis to official inequality measures. In a later stage, age-specific Gini coefficients are added to the ESS 5 data set. Here, a measure of generalized trust emerges from the survey question phrased “...*generally speaking, would you say that most people can be trusted, or that you can’t be too careful in dealing with people?*”, with a response scale from 0 (“You can’t be too careful”) to 10 (“Most people can be trusted”). Lastly, the ESS 5 supplies us with the individual’s age, other personal characteristics (sex, marital and employment status, level of education etc.) as additional control variables. All applied variables are summarized in Table 1.

**Table 1:** Summary of characteristics: model variables are distributed on a national (macro), age-specific (mezzo), and individual (micro) level. *Source* EU-SILC 2009, ESS 5, and own calculations

Variable	Observations	Mean/average	Max	Min
National Gini	22	0.339	0.390	0.261
Age group Gini	1429	0.327	0.411	0.237
[0–10] Generalized trust	22,089	4.89	10	0
[0/1] Gender: male	22,089	49.20 %	–	–
Age	22,089	49.77	81	17
Years of education	22,089	12.26	45	0
Work status				
Paid work	22,089	40.0 %	–	–
Education	22,089	10.7 %	–	–
Unemployed	22,089	7.5 %	–	–
Retired	22,089	3.3 %	–	–
Housework	22,089	4.7 %	–	–
Other	22,089	4.2 %	–	–
Marital status				
Married/civil union	22,089	40.5 %	–	–
Separated/divorced	22,089	10.3 %	–	–
Widowed	22,089	10.4 %	–	–
Never married	22,089	38.8 %	–	–
Children living at home	22,089	51.6 %	–	–
Born in country	22,089	92.6 %	–	–

## Method

The method of this work can be basically drawn in three distinct steps. First, age group measures of income inequality are calculated. Then second, with the use of a mixed effect hierarchical ordered logit model with country and age clusters, levels of generalized trust are explained by country Ginis and age-specific Ginis. Individual covariates are choosen according to the reasoning of selected previous investigations. Possible influences on how much people trust each other have been discussed widely in the socio-economic literature in the last 15 years. Six of the most cited investigations on trust^3^ with multilevel models serve as a pool for the selection of micro-level characteristics. Some features have been used more frequently than others and studies differ in their regional focus and applied metric. Lastly, standard income inequality and age-specific income inequality are cotrasted with respect to their effects on generalized trust.

### Income Inequality by Age

The key measure of this analysis is an age-specific Gini coefficient. The coefficient is calculated for each age, taking into consideration individuals within an 11-year age-span (individuals 5 years younger and 5 years older than the reference age). In this context several smaller age-spans (3, 5, 7, 9 years) have been tested—with no significant change of results. Results lose significance once a 1-year-span is chosen.^4^ A formula often advocated for its easy applicability is used (Milanovic 1997):

1 $$\begin{aligned} G_{a}&= \frac{1}{\sqrt{3}} \frac{\sigma _{y_{a}}}{\mu _{a}} \rho (y_{a},r_{y_{a}}), \nonumber \\ a&= 18,\ldots 80 \end{aligned}$$

Here, $G_{a}$ the stands for the Gini index of individuals of age *a*. $\rho (y_{a},r_{y_{a}})$ is the covariance between income $y_{a}$ and the rank of all individuals according to their income $r_{y_{a}}$ within one age group. $\sigma _{y_{a}}$ denotes the variance of income in one group and $\mu _{a}$ the mean income in this age group. For instance, the age-specific Gini $G_{30}$ would refer to the income inequality of all individuals aged between 25 and 35 in one county. For the less complex case, only groups of similar age are considered as basis for the calculation. Table 2 compares the national Gini with the age-specific Ginis. The correlation coefficient between the country- and the age-specific Ginis of 0.65 is more than moderate butl not large enough to assume identity of the two measures.

**Table 2:** Country characteristics: for 22 European countries, national and age-specific Ginis are compared. *Source* EU-SILC 2009, ESS 5, and own calculations

Country	Observations	National Gini	Age group Gini		
			Min	Mean	Max
NO	849	0.26	0.24	0.26	0.29
NL	1057	0.27	0.25	0.27	0.29
DK	902	0.27	0.24	0.27	0.29
SE	901	0.30	0.26	0.29	0.32
HU	787	0.30	0.29	0.30	0.31
SI	476	0.31	0.25	0.31	0.37
CZ	1282	0.32	0.31	0.32	0.33
FR	992	0.33	0.31	0.33	0.34
SK	793	0.33	0.31	0.33	0.35
EE	936	0.33	0.30	0.33	0.34
BE	913	0.33	0.32	0.33	0.35
DE	1870	0.34	0.32	0.33	0.35
PL	712	0.35	0.33	0.34	0.36
CH	838	0.35	0.33	0.35	0.36
IE	1383	0.35	0.33	0.35	0.38
ES	947	0.35	0.32	0.34	0.36
UK	1317	0.38	0.35	0.38	0.40
CY	494	0.38	0.35	0.37	0.40
GR	1415	0.39	0.36	0.38	0.40
BG	1314	0.39	0.34	0.39	0.41
LT	739	0.39	0.37	0.39	0.40
PT	1172	0.39	0.35	0.39	0.41

Some countries show a sizeable variance in inequalities across age groups

### Explaining Trust

The model for the examination of the reference-specific linkage between income inequality and generalized trust can be described in the following way:

2 $$\begin{aligned} P(Trust_{i}&= Trust|x) = \alpha _{c,a} + \alpha _{c} + \beta _{c}\cdot Gini_{c} + \delta _{c,a}\cdot AgeGini_{c,a} + \gamma _{c,a,i}\cdot Individuals_{c,a,i} + \epsilon _{c,a,i} \end{aligned}$$

**Fig. 2:**
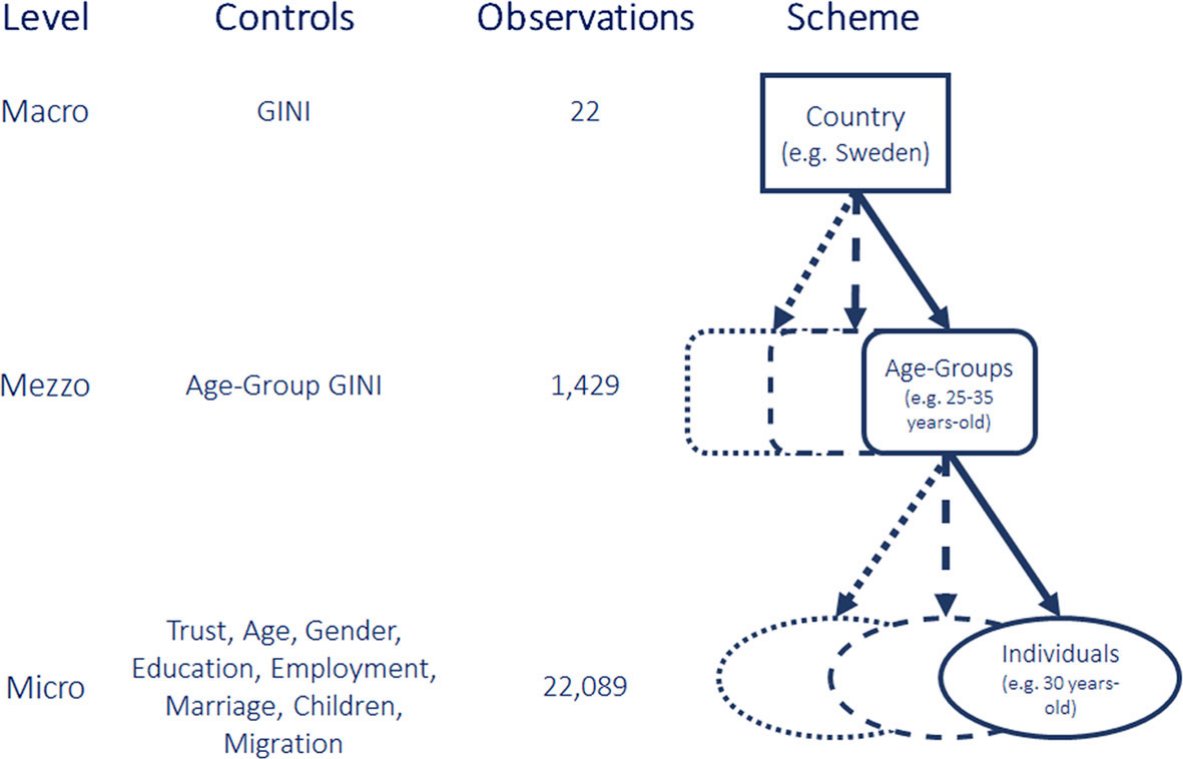
Illustration of model: the three-dimnesional model segregates the sample into macro- (countries), mezzo- (age groups), and micro-levels (individuals), which are nested within each other. *Source* EU-SILC 2009, ESS 5, and own calculations

Trust, measured on a scale from 0 to 10 on the individual (*i*) level is explained by the *AgeGini* coefficient, specific for each age group (*a*). In addition, the national Gini, on a country level (*c*) is taken into account. Since the dependent variable is ordinal and individual characteristics are nested within age groups which are again nested within countries, a hierarchical model structure is applied. Figure 2 illustrates the structure of the model with its micro-, mezzo- and macro-level.

As explained in the previous paragraph, in this way, country-specific and age-specific fixed effects are allowed. The age-specific Gini model is compared with a baseline model containing the national Gini. At the last model stage, the interaction of both Ginis is considered.

### Model Specification

**Fig. 3:**
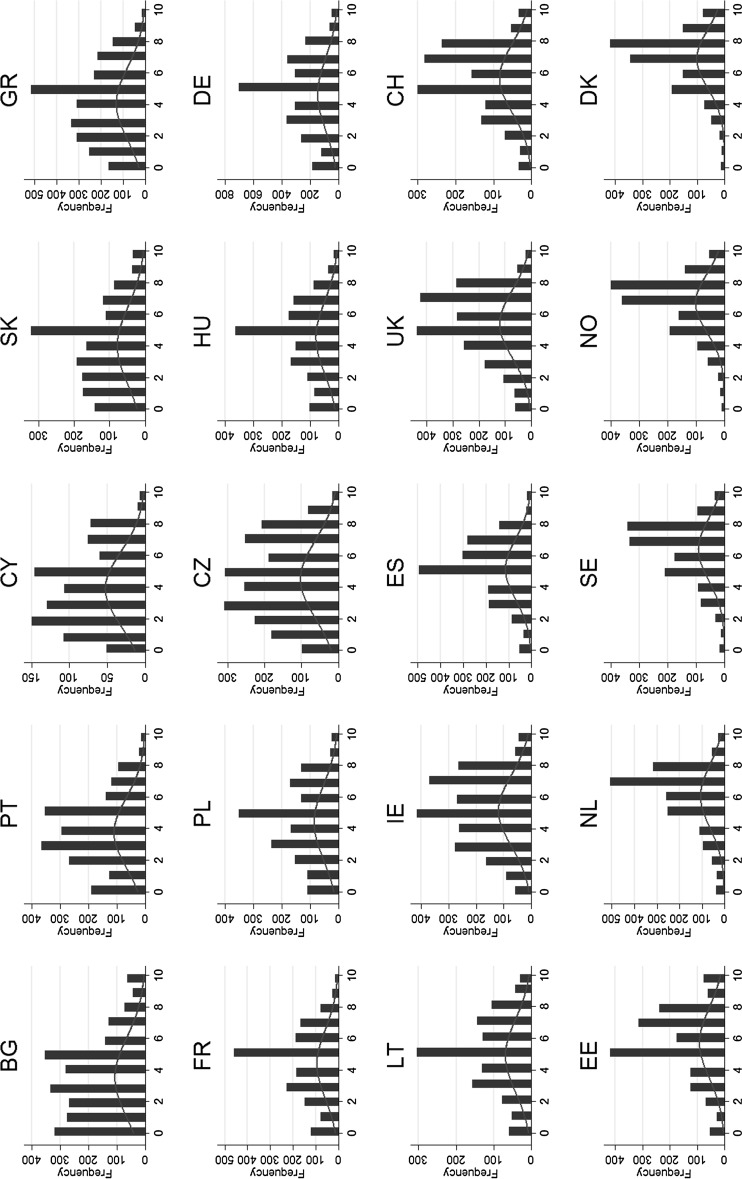
Distribution of Trust Across Countries: the distribution of generalized trust across countries suggests an ordinal logit model environment. *Source* EU-SILC 2009, ESS 5, and own calculations

Figure 3 responses to the generalized trust question are displayed in histograms per country. The distribution of the trust outcome in general appears to be well captured with a logit distribution.

For most countries patterns of trust are almost symmetrically distributed, but with thick tails. Therefore the applied model with an ordinal outcome assumes a logit distribution. Secondly, it can be noticed that the shape of the distribution varies significantly across countries. We can observe a left shift of the distribution in low trust countries like Bulgaria and Cyprus, a symmetric distribution in countries like Germany and Lithuania, and a right-skewed pattern in high trust countries like Norway and Denmark. In the model, country fixed effects are considered in order to ensure a more precise estimation. With regard to the structure and distribution of the data, a hierarchical generalized linear ordinal logit model with an assumed logit distribution of trust is applied.^5^

Having specified the selection of the model structure according to the distribution of the outcome variable, careful attention is drawn to the selection of valid aggregated and individual characteristics explaining trust. The discussion about possible explanatory factors on a theoretical ground is necessary in order to build a reliable model environment. However, conceptual considerations could become arbitrary if not validated with a sound selection procedure. The limited sample size of 22 countries does not allow for any further first level controls, but national Gini. All controls described in the following, which have been chosen by theoretical reflection in previous works (Steijn and Lancee 2011; Leigh 2006b; Uslaner and Brown 2005; Alesina et al. 2004), are evaluated on the basis of their explanatory power. Table 3 summarized these works.

**Table 3:** Variable selection: the six investigations on the determinants of generalized trust have applying different controls *Sources* Alesina and La Ferrara (2002), Hooghe et al. (2009), Leigh (2006b), Leigh (2006a), Marschall and Stolle (2004), Rothstein and Stolle (2008). *Source* EU-SILC 2009, ESS 5, and own calculations

	Alesina and La Ferrara (2002)	Hooghe et al. (2009)	Leigh (2006a)	Leigh (2006b)	Marschall and Stolle (2004)	Rothstein and Stolle (2008)	ESS5 (2010)	Selection
Individual variables								
Age	$\checkmark$	$\checkmark$	$\checkmark$	$\checkmark$	$\checkmark$	$\checkmark$	$\checkmark$	$\checkmark$
Gender	$\checkmark$	$\checkmark$	$\checkmark$	$\checkmark$	$\checkmark$	$\checkmark$	$\checkmark$	$\checkmark$
Education	$\checkmark$	$\checkmark$	$\checkmark$	$\checkmark$	$\checkmark$	$\checkmark$	$\checkmark$	$\checkmark$
Employment status	$\checkmark$	$\checkmark$	$\checkmark$			$\checkmark$	$\checkmark$	$\checkmark$
Marital status	$\checkmark$			$\checkmark$		$\checkmark$	$\checkmark$	$\checkmark$
Children	$\checkmark$				$\checkmark$		$\checkmark$	$\checkmark$
Income	$\checkmark$						$\checkmark$	
Religion	$\checkmark$						$\checkmark$	
Life satisfaction						$\checkmark$	$\checkmark$	
Race	$\checkmark$						na	
Migrant background		$\checkmark$	$\checkmark$				$\checkmark$	$\checkmark$
Church attendance		$\checkmark$					$\checkmark$	
Commuting			$\checkmark$				na	
Length of residence			$\checkmark$		$\checkmark$		na	
Type of residence							$\checkmark$	
Interracial contact					$\checkmark$		na	
Trauma in past	$\checkmark$						na	
Association membership							$\checkmark$	
Rating of police						$\checkmark$	$\checkmark$	
Rating of justice system						$\checkmark$	$\checkmark$	
Rating of political system		$\checkmark$					$\checkmark$	
Neighbourhood problems					$\checkmark$		na	
Model specification								
Region	USA	Europe	Australia	World	USA	World	–	Europe
Level of aggregation	Federal states	Countries	Neighbour-hoods	Countries	Neighbour-hoods	Countries	–	Countries and age groups
Number of groups	51	20	692	59	15	71	–	22 and 1429
Number of observations	7325	36,697	6500	82,778	902	84,006	–	22,089
Micro-level model	Yes	No	No	Yes	No	Yes	–	Yes
Macro-level model	Yes	No	No	Yes	No	Yes	–	Yes
Mixed model	No	Yes	Yes	No	Yes	Yes	–	Yes

Age, gender, education, employment, marriage, children, and origin are commonly applied

On an individual level, a collection of commonly mentioned indicators is conducted. All controls, which have been used at least twice in previous contributions are selected, if available in the ESS dataset. The final list includes namely: age, gender, years of education, employment status, marital status, having children, and born in the country.

## Results

### Inequality by Age

**Fig. 4:**
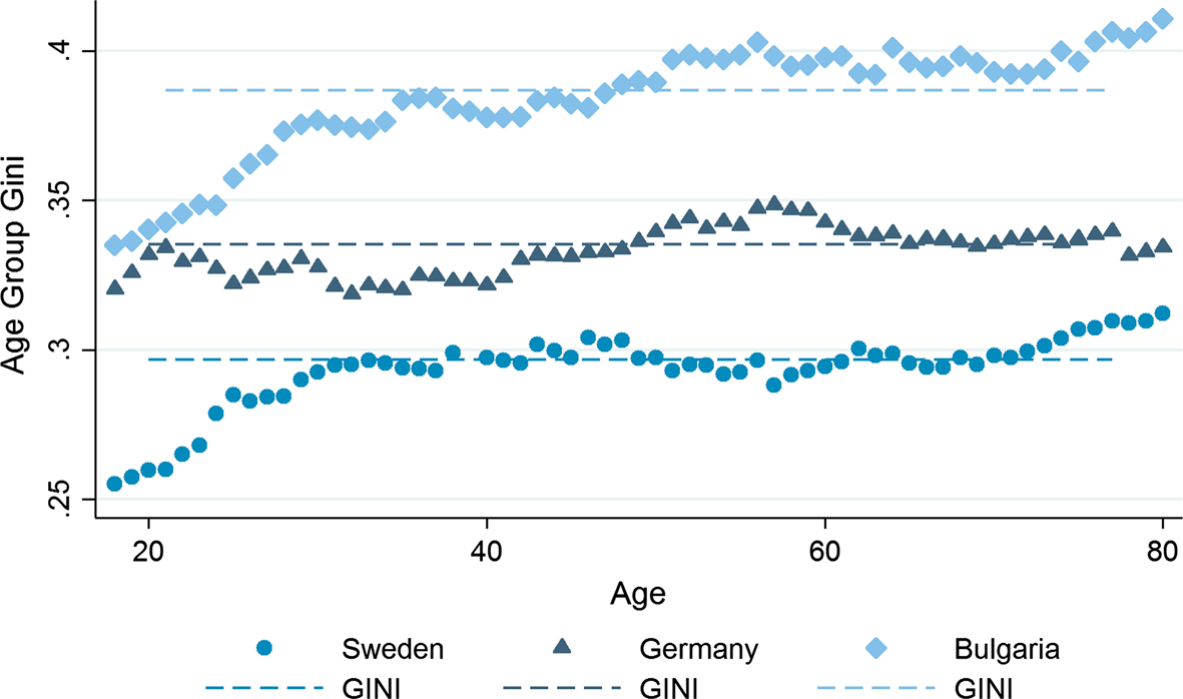
Age group Ginis for three selected countries: the example of Sweden, Germany, and Bulgaria shows that age-specific imbalances remain undetected by national measures of inequality. *Source* EU-SILC 2009, ESS 5, and own calculations

Socio-economic referencing has been shown to transmit between inequality and trust. The application of measures of inequality on a national level overlooks income imbalances within certain social groups. It is imparative to examine subnational inequality in order to better understand the link between inequality and trust. This analysis fragments society by age and creates Gini coefficients for 11 years age spans from the ages of 18–80. Figure 4 illustrates the age group Ginis for the example of three countries with different levels of national income inequality, namely Sweden (low inequality), Germany (medium inequality), and Bulgaria (high inequality).

Even though the national level of Gini is significantly different across all three countries, age specific measures reveal how disperse income inequality is within the socities. Starting with a low inequality example, Sweden, it can be noticed that income imbalances increase with age for the first 10 years of the sample populatiopn. Imbalances remain fairly stable and peak again for the ten last age groups. The lowest age group gini in Sweden is 0.255 and the highest level at 0.33. Sweden’s highest level of age-specific inequality almost reaches the lowest values of the German age group inequality. Here, income inequality, on the other hand, develops relatively stable across ages. Imbalances peak slightly around the age of 57. Bulgaria, lastly, shows by far the largest variance in inequality. For early ages, the levels of Gini still lie around the German national average of 0.34. However, across age, inequality increases starkly and reaches its highest value for the last age group with a value of 0.41. The first results indicate that age-specific income inequality is not necessarly reflected by national measures. If referencing based on socio-economic characteristics, like age, is an important determinant of generalized trust, age-specific measures of inequality need to be considered for the analysis of the inequality-trust-linkage.

### Patterns Across Age and Education

**Fig. 5:**
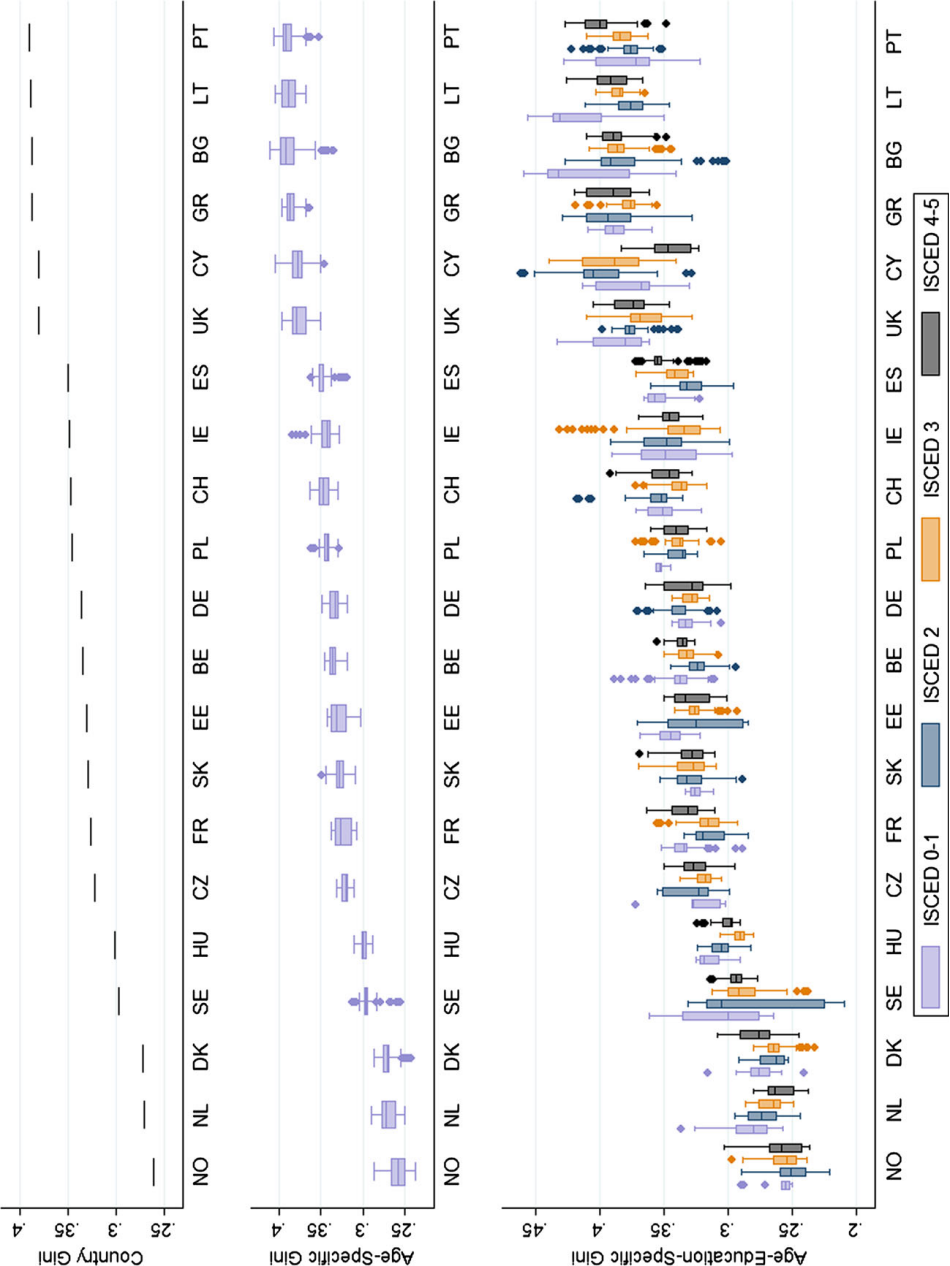
Gini coefficients ordered by national Gini: for countries with higher levels of inequality, dispersion along age and educational groups seems to be larger. *Source* EU-SILC 2009, ESS 5, and own calculations

**Fig. 6:**
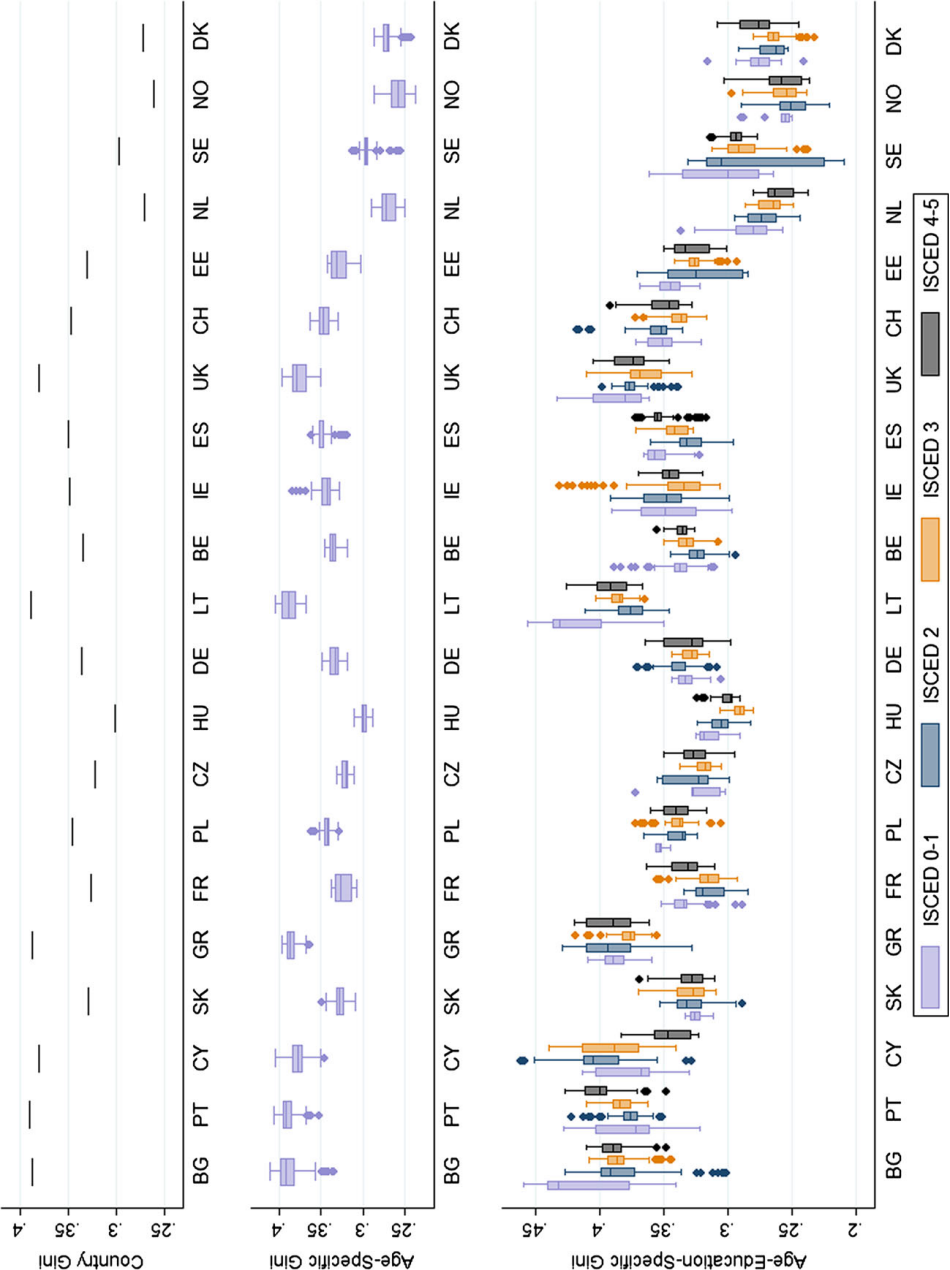
Gini coefficients ordered by national trust: anegative association between inequality and trust becomes apparent. *Source* EU-SILC 2009, ESS 5, and own calculations

In addition to age, the socio-economic reference group can be further defined by education. Figures 5 and 6 display different measures of income inequality for 21 European countries.^6^

The graphical display is structured as followed from top to bottom. First, the country level Gini is shown. Secondly, in the middle of the graph, distributions of age-specific Gini coefficients (age span of 11 years) are display. In the lowest part of the graph, the distribution of age- and education-specific Ginis is depicted. It is important to notice that the third part does not just separate the information of the middle section by educational attainment. The measures of inequality, in the lowest part, are calculated from different populations. Here, only individuals within one age and educational group are considered. Figure 6 relies on the same information as Fig. 5, but countries are ordered by national level trust instead of national Gini. In general, it can be noticed that countries with a low level of national trust, like Bulgaria, Portugal and Cyprus, have a high level of general and age-education-specific inequality. While high trust countries like Sweden, Norway, Denmark or the Netherlands, show low levels of reference-specific inequality on the other side.

The positive association between age-specific inequality and national inequality is indicated by the upward trend of the distributions’ means with higher level of national Gini. Additionally, it can be noticed that the variance of age-specific inequality is higher for countries of high national inequality. An exception, in terms of variation of inequality, is Sweden, where variation in age-education-specific Ginis in the first two educational groups is large, though the level of national inequality is low. Since economic benchmarking within groups of similar age and education is suspected to play an important role for mutual confidence, this surprisingly rich spectrum of socio-specific inequality needs to be considered.

The significant within-country variation of income inequality is not captured the national inequality measure in the first row. While Scandinavians are—as expected—societies with low income imbalances, countries like Greece, Bulgaria and Portugal appear to have relatively skewed income distributions. With the introduction of age- and education-specific Gini coefficients, additional patterns of income inequality emerge. At first sight, sizable difference in the age-specific imbalances the within countries and across education levels are present. As an example, in the Netherlands, a country with egalitarian roots and known for its low level of average inequality, age-specific Ginis decrease with level of education. In Portugal, the country with the sample’s highest level of general inequality, age-specific imbalances seem to increase with educational class. Societies like Germany or Poland, middle-ranked in Europe’s score of income inequalities, do not show any distinct differences in age-specific inequality across education. Another interesting case is the example of Lithuania. Looking at Fig. 6 in which countries are ordered by trust, the Baltic society seems to stick out of the average pattern with a relatively high level of general inequality. Though, the split-up by age and education shows that high levels of country inequality are mainly driven by income imbalances in the lowest educational groups.

### Which Inequality Really Matters?

 As seen previously, high income inequality is associated with low levels of trust. But does inequality hinder trust? The relationship is sketched by two concepts, stratification and perception. For evaluating the *perception effect* of inequality it is argued that age and education could be interpreted as the lowest common. Table 4 exhibits the effects of the various Ginis (and their interaction) on trust, while all mentioned controls are applied.

**Table 4:** Generalized Trust Explained by Inequality: trust is negatively associated with both national and age group inequality *Source* EU-SILC 2009, ESS 5, and own calculations

VARIABLES	(1)	(2)	(3)	(4)
	[0–10] Generalized trust			
Country Gini	−12.17***		−10.41***	−1.16
	(0.57)		(0.42)	(1.51)
Age group Gini		−1.16***	−0.41**	2.53
		(0.21)	(0.23)	(1.63)
Country Gini × age group Gini				−8.37*
				(4.65)
Age	−0.02***	−0.01	−0.01**	−0.01**
	(0.00)	(0.01)	(0.01)	(0.01)
Age^2^: 1000	0.23***	0.12**	0.18***	0.17***
	(0.04)	(0.06)	(0.06)	(0.06)
[0/1] Gender: male	0.05**	0.06**	0.06**	0.06**
	(0.02)	(0.02)	(0.02)	(0.02)
Years of full-time education completed	0.07***	0.08***	0.08***	0.08***
	(0.00)	(0.00)	(0.00)	(0.00)
Work status (ref. employed)				
In education	0.18***	0.17***	0.17***	0.19***
	(0.05)	(0.05)	(0.05)	(0.05)
Unemployed	−0.24***	−0.21***	−0.21***	−0.20***
	(0.05)	(0.05)	(0.05)	(0.05)
Retired	−0.14***	−0.11**	−0.13***	−0.13***
	(0.04)	(0.05)	(0.05)	(0.05)
Housework	−0.08	−0.06	−0.04	−0.05
	(0.06)	(0.06)	(0.06)	(0.06)
Other (sick, community service, etc.)	−0.20***	−0.21***	−0.21***	−0.22***
	(0.06)	(0.06)	(0.06)	(0.06)
Marital status (ref. Married/Legal Union)				
Separated/divorced	−0.10**	−0.11***	−0.11***	−0.11***
	(0.04)	(0.04)	(0.04)	(0.04)
Widowed	−0.09**	−0.08*	−0.10**	−0.09*
	(0.05)	(0.04)	(0.04)	(0.04)
Never married	−0.04	−0.02		−0.01
	(0.04)	(0.04)	(0.04)	(0.04)
[0/1] Ever had children living in household	−0.02	−0.01	−0.02	−0.02
	(0.04)	(0.04)	(0.04)	(0.04)
[0/1] Born in country	0.12***	0.13***	0.10**	0.11**
	(0.05)	(0.05)	(0.05)	(0.05)
Countries	22	22	22	22
Age groups	1429	1429	1429	1429
Individuals	22,089	22,089	22,089	22,089
AIC	4.295	4.294	4.291	4.291

Model precision increases with the application of age-specific measures

Standard errors in parentheses

*** *p* < 0.01; ** *p* <  0.05; * *p* <  0.1

In column (1), the national Gini shows a strong negative association with trust, as expected in accordance with the literature. All individual controls show results, which a similar to previous investigations. Generalized trust decreases with age and increases with education. While man are more trusting than women, on average, individuals in education and with stable employment and partnerships likewise show higher levels of confidence. The same is true for native nationals. In model (2), national Gini is substituted by age-specific Gini. Overall, the results are similar. Age-specific inequality is likewise negatively associated with generalized trust. When both measures of inequality are taken into account in model (3), age-specific inequalities still show a negative association with generalized trust. The within country variation of income inequality and influence on trust is not captured by national Ginis alone. In model (4) lastly, the interaction between national and age-specific Gini is examined. Here, both measures seem to reinforce each other. Generalized trust is significantly lower where both national and age-specific inequality is high. Comparing the model lines, AIC indicates that model accuracy can be improved with the use of the age-specific Gini measurement.

## Conclusion

As seen in previous contributions, high income inequality is associated with low levels of trust. But does the concept of national income distribution convincingly describe the essence of why inequality hinders trust? The relationship is sketched by two concepts, stratification and perception. The economic stratification of society leads to the emergence of distinct spheres of everyday life. Segregated housing and income-dependent school systems are vivid examples of how inequality can diminish the ground of common trust building. The argument of perception, based on subjective believes, is more difficult to illustrate. On the one hand, it is less likely to keep up a relationship, rich in trust, with others of who we think to share only little similarities. On the other hand, it is the believe that society is marked by unrightful differences which diminishes our faith in one another.

Previous studies have claimed to capture the effects of both stratification and perception with the use of one inequality indicator. Though, if one believes that the comparison with others is key to understand how inequality influences trust, the concept of economic reference groups should be described accordingly. Instead to comparing one’s own economic well-being to the rest of the society, it is much more likely, that we chose a reference group to which a meaningful comparison is possible, in the first place. For an evaluation of the *perception effect* of inequality, *“our family Jones”*, to use the term of social class benchmarking, still needs to be defined. In the analyzed case, it is argued that age and education could be interpreted as the lowest common. The findings rather support age as a reference criterion. The results have four main indications. First, inequality in general diminishes mutual confidence. Second, both low and high inequality countries inherit sizeable age-specific income imbalances. Third, empirical results suggest that the concept of age-specific inequality enriches the theory of economic imbalances as a treat to generalized trust. In interaction with general imbalances, age-specific inequality reinforces effects of trust. Where ever age-specific inequality is high, levels of trust are even lower if general inequality is high too.

### The Inequality Within Inequality

The common conclusion of previous works that high inequality is accompanied by low levels of trust is supported. Indeed, in contrast to previous studies skeptical about the impact of income inequality on trust, the presented work consolidates the influence of inequality even when regarding a large set of other economic and personal characteristics at the same time. This finding appears to be robust, but economic stratification of society is only one aspect of how inequality diminishes trust.

The results of this study underline that the perception of income imbalances in Europe plays an important role in explaining generalized trust. Economic comparison with other members of society impacts on how much we trust each other. The question to whom we compare our standards of living is relevant and the concept of an adequate socio-economic reference group needs to be considered. Age and education, among many other possible characteristics, are use to build examples of socio-economic groups, within which measure of income inequality are calculated. The introduction of these sub-categories of inequality has uncovered that income imbalances within European societies vary to a sizable extent across age and educational groups. As one, of many, illustrative examples; in Sweden, a society with low levels of national inequality, the variation of age-specific inequality is stricingly different comparing age groups and levels of education. Differences range from low levels of group-specific inequality around 0.2 up to values above 0.35. The fact that socio-economic reference determines our mutual confidence underlines the importance to consider this spread.

The results have illustrated that the aspect of social referencing matters for determining generalized trust. Additionally we see that the boundaries of economic benchmarking are rather set by age alone than by age and education. The large spread of age- and education-specific income imbalances in some European countries should alert researchers as well as policy makers, who have so far been mostly relying on ample measure of income inequality. The results presented here show that also outside the context of inequality and trust, a more refined depiction of within country imbalances of income is advisable. In the specific context of trust and inequality, age-specific inequality is no substitute for actual inequality in modeling trust, but rather a complement. In environemnts where inequality per se is high, negative effect of socio-specific inequality on trust are even more intense.

### Future Research

The presented work has underlined the negative relationship between income inequality and generalized trust. Furthermore, it encourages researchers in this field to have a closer look at patterns of inequality beyond the known standard aggregated measures. Age-specific inequality is negatively associated with trust, too. Their diminishing effect on trust might be overlooked in countries with an apparently low level of income inequality. Still without a timing dimension, associations will not give any disclosure on causal links. Only by following patterns of inequality and trust over time in future studies, one can find evidence to challenge the debate about reverse causality between fragmentation and trust.

The successful application of subnational-aggregates should foster future refinements in the analysis of generalized trust and fragmentation. Just like age-specific imbalances are not in line with aggregates of inequality, a sizable regional variation of trust, inequality, and other measures of fragmentation can be expected. The analysis of the inequality-trust-linkage on a regional level is surely an valuable and promising topic of investigation.

## Appendix

**Description of Data and Variables**

European Union Statistics on Income and Living Conditions (EUSILC UDB 2010—version 5 of March 2014); countries covered: AT 10,318; BE 7810; BG 15,441; CH 11,826; CY 4829; CZ 11,758; DE 18,588; DK 14,499; EE 11,209; EL 9486; ES 20,814; FI 17,863; FR 21,904; HU 13,928; IE 10,043; IS 7914; IT 24,758; LT 12,452; LU 5,754; LV 14,413; MT 6623; NL 21,038; NO 11,671; PL 19,709; PT 7513; RO 10,017; SE 10,110; SI 9364; SK 8180; UK 14,413.

European Social Survey 5 (ESS 5)—2010; countries covered: BE 1644; BG 2249; CH 1429; CY 906; CZ 2110; DE 2882; DK 1475; EE 1659; ES 1778; FR 1657; GR 2504; HU 1415; IE 2246; LT 1215; NL 1723; NO 1487; PL 1581; PT 1957; SE 1406; SI 1094; SK 1537; UK 2145.

## Footnotes

1 In this work, the terminology inequality stands for income inequality.

2 In English-speaking countries “Keeping up with the Joneses” is an idiom for a comparison to one’s neighbours as a social class benchmark. It has been adapted by economic literature in order to describe socio-economic referencing.

3
Alesina and La Ferrara (2002), Marschall and Stolle (2004), Leigh (2006a, b), Rothstein and Stolle (2008) and Hooghe et al. (2009).

4 For a descriptive analysis, education in addition to age is taken into account. This measure, however, is not applied in the inferential investigation, since the sample populations by age and education are too small for several countries. For the calculation of the age-education-specific Gini, only individuals within the same age group and with a similar level of educational attainment (ISCED 0–1, ISCED 2, ISCED 3, and ISCED 4–5) are considered.

5 The statistical software STATA MP 13 employs the *meglm* command for multilevel mixed-effects generalized linear models. The model commands for distribution and link are choosen as *famliy(ordinal)* and *link(logit)*.

6 The sample size for Luxembourg is too small in order to perform the fragmentation with education.
